# Rapid Identification of Chinese Hamster Ovary Cell Apoptosis and Its Potential Role in Process Robustness Assessment

**DOI:** 10.3390/bioengineering10030357

**Published:** 2023-03-14

**Authors:** Shang Xiao, Qiang Li, Jinlong Jiang, Chengxiao Huo, Hao Chen, Meijin Guo

**Affiliations:** 1 State Key Laboratory of Bioreactor Engineering, East China University of Science and Technology, Shanghai 200237, China; shang.xiao@lyvgen.com (S.X.); novelleeqiang@126.com (Q.L.);; 2 Shanghai Biological Products Research Institute Co., Ltd., Shanghai 200051, China; huochengxiao@sinopharm.com (C.H.); chenhao20@sinopharm.com (H.C.)

**Keywords:** CHO, apoptosis, glucose withdrawal, robustness, process characterization

## Abstract

Currently, the assessment of process robustness is often time-consuming, labor-intensive, and material-intensive using process characterization studies. Therefore, a simple and time-saving method is highly needed for the biopharmaceutical industry. Apoptosis is responsible for 80% of Chinese hamster ovary (CHO) cell deaths and affects the robustness of the cell culture process. This study’s results showed that a more robust process can support cells to tolerate apoptosis for a longer time, suggesting that the robustness of the process could be judged by the ability of cells to resist apoptosis. Therefore, it is necessary to establish a rapid method to detect the apoptosis of CHO cells. In trying to establish a new method for detecting apoptosis in large-scale cell cultures, glucose withdrawal was studied, and the results showed that CHO cells began to apoptose after glucose was consumed. Then, the concentration of extracellular potassium increased, and a prolongation of apoptosis time was observed. Further study results showed that the process with poor robustness was associated with a higher proportion of apoptosis and extracellular potassium concentration, so potassium could be used as a biochemical index of apoptosis. The strategy we present may be used to expedite the assessment of process robustness to obtain a robust cell culture process for other biologics.

## 1. Introduction

CHO cells are the most widely used mammalian host cells in large-scale biopharmaceutical production, owing to the similar post-translational modifications in protein expression to those in humans and safe therapeutic protein production [1,2]. In 2018, 57 of 68 commercially available monoclonal antibodies were produced in CHO cells and generated an approximately USD 107 billion market [3]. In response to rapidly growing market demands and competition [4], CHO cell culture processes should be optimized to reduce production costs and improve process robustness [5]. At present, many studies have reported cases of improving expression through process optimization [6,7], but studies on how to evaluate the robustness of the process are rarely reported. Good process robustness is required by engineers and regulators [8,9]. To demonstrate the process robustness, process characterization should be performed. Process characterization is associated with high workload and is time-consuming [10,11].

Apoptosis and lactate are important factors that cause process instability. Figueroa et al. reported that apoptosis was a major cause of death of CHO cells [12]. Many previous studies have shown that many factors in the process could lead to apoptosis, such as shear stress, nutritional deficiency, temperature, osmolality and so on [13,14]. Therefore, the detection of apoptosis in CHO cell culture is critical to reduce the process failure caused by apoptosis, whether it is cell line engineering or the optimization of production process conditions. Unfortunately, no simple method for the rapid characterization of apoptosis has been reported.

Lactate production is a huge challenge in CHO cell culture and negatively affects the process robustness, protein expression, and quality [15,16]. Lactate is produced by the glycolysis of glucose [17,18], and its accumulation can be minimized in CHO cell culture by limiting the glucose concentration [12,19]. Glucose withdrawal is an important tool to reduce lactate accumulation, and most previous studies on glucose withdrawal focused on inducing a shift from lactate production to consumption [15,20]. Glucose also plays a critical role in CHO cell growth and metabolism, which provides cells with necessary adenosine triphosphate (ATP) via two pathways: glycolytic fermentation and oxidative phosphorylation. It also provides cells with various metabolic substrates via the pentose phosphate pathway [21]. Fan et al. reported that glucose withdrawal could affect the expression and N-glycosylation of monoclonal antibodies in the fed-batch CHO cell culture [22]. Therefore, glucose is a good tool for studying the characteristics of CHO cells.

In order to identify methods to quickly characterize the apoptosis and robustness of the CHO cell culture process, a small-scale culture system was used to represent the typical manufacturing-scale serum-free fed-batch process, and two different fed-batch processes were used in this study. Glucose withdrawal was used as a method to induce apoptosis, and the timepoints of glucose withdrawal were carefully chosen to coincide with the exponential and stationary phases. Furthermore, the tolerance of CHO to apoptosis under different processes was studied to evaluate the relationship between apoptosis and process stability.

## 2. Materials and Methods

### 2.1. Cell Lines and Fed-Batch Process

An antibody-producing suspension CHO-K1 cell line was used as the model cell line. All basal media and feeds used in this study were proprietary, chemically defined, and serum-free. Before the fed-batch culture, cell lines were passaged every 4 days at a density of 3 × 10^5^ cells/mL in a humidified incubator maintained at 36.5 °C and 5% CO_2_ with shaking at 120 rpm.

Experiments were performed in 3 L bioreactors (Applikon, Delft, Netherlands) with an initial culture volume of 1.5 L, and 250 mL shake flasks with an initial culture volume of 50 mL. In this study, two processes were performed, which only differed in incubation temperature. The temperature of process 1 was shifted to 33 °C on day 5, while that of process 2 was maintained at 36.5 °C.

The basal media and feeding media used in this study were chemically defined serum-free media. The basal medium was Dynamis, which was purchased from Thermofisher. The feeding media were FM01A and FM01B, which were developed by our laboratory but produced by Merck Sigma (Nantong, China). From the third day, FM01A added 3% of the initial culture weight every day and each FM01B supplement was 1/10 of FM01A. Both FM01A and FM01B did not contain glucose. Glucose was controlled at 6 g/L by feeding 400 g/L of glucose solution daily.

### 2.2. Cell Density, Metabolite and Amino Acid Analyses

Cell culture samples were obtained from each bioreactor and shake flask, and the viable cell density (VCD), viability, and cell diameter were measured in an automated cell-counting device (Vi-CELL, Beckman, Brea, CA, USA) by trypan blue staining. Lactate, glucose, and potassium ion levels were monitored using RAPIDPoint 500 (SIEMENS, Berlin, Germany). When lactate and glucose levels were below the detection limit, they were defaulted to 0.

Supernatant samples were stored at −80 °C. At the end of the experiments, frozen cell-free supernatant samples were thawed and collectively submitted for the yield analysis with high-performance liquid chromatography (HPLC; HP1100, Agilent, Palo Alto, CA, USA).

Amino acids were analyzed by ultra-high performance liquid chromatography (UPLC, Waters, Milford, MA, USA). Firstly, the standard and test samples of amino acids were labeled with Waters AccQ-Tag derivative kit (item number: 186003836, Waters, Milford, MA, USA), then the labeled amino acids were separated by reversed phase chromatography column (1.7 um, 2.1 × 100 mm) and detected by an ultraviolet detector at the detection wavelength of 260 nm. The contents of different kinds of amino acids in the sample were determined by a standard curve external standard method.

### 2.3. Antibody Titer Analysis

After centrifugation with a centrifugal force of 4000 g, samples were injected into the HPLC system (Agilent, Palo Alto, CA, USA) equipped with UV detection at 280 nm. The column was TSKgel Protein A-5PW 4.6 × 35 mm, 20 μm (Tosoh, Shanghai, China). The flow rate was 1 mL/min. The gradient method using mobile phase 50 mM sodium phosphate/150 mM sodium chloride and 100 mM glycine/150 mM sodium chloride was used to elute each sample every 8.0 min [23].

### 2.4. Physicochemical Analysis

Cell culture bulk was collected and purified by a protein A column.

For size variant analysis, the samples were analyzed by a TSK G3000SWXL column 7.8 × 300 mm, 5 μm (Tosoh, Shanghai, China) with a mobile phase buffer (50 mM NaH_2_PO_4_, 250 mM NaCl, pH 6.8) at a constant flow rate of 0.5 mL/min.

For size variant analysis, CE-SDS was performed under nonreducing conditions for the analysis of purity/impurities. A Beckman Coulter, PA 800 capillary electrophoresis system was used, with an effective length of 30.2 cm and a 50 mm I.D. bare-fused silica capillary.

For charge variant analysis, the samples were analyzed by Propac WCX10 4 × 250 mm, 5 μm (Thermo, Waltham, MA, USA). Gradient elution was performed at a constant flow rate of 0.8 mL/min.

For oligosaccharide profile analysis, N-linked glycans were first enzymatically released from the antibody with peptide-N-glycosidase F (pNGase F), labeled with 2-aminobenzamide, and subsequently analyzed by ultra-performance liquid chromatography (UPLC) with fluorescence detection.

### 2.5. Apoptosis Analysis

Moreover, 1 × 10^5^ cells were re-suspended in 200 μL binding buffer, 4 μL 0.5 mg/mL PI, and 2 μL Annexin V-FITC solution was added, incubated at room temperature for 15 min, and detected by flow cytometry. The maximum excitation light of Annexin V-FITC was 488 nm, and the emission light was 520 nm. The maximum excitation light of PI was 561 nm, and the emission light was 617 nm. However, both of them could be excited by a 488 nm laser.

### 2.6. Statistical Analysis

Statistical analyses were performed using SPSS 19 software. Data are expressed as mean ± standard error. The data shown in the figures are of experiments performed in triplicate. The *t*-test was performed for comparisons. A *p*-value < 0.05 was considered to indicate statistical significance.

## 3. Results

### 3.1. Apoptosis of CHO Cells Induced by Glucose Withdrawal

**Figure 1:**
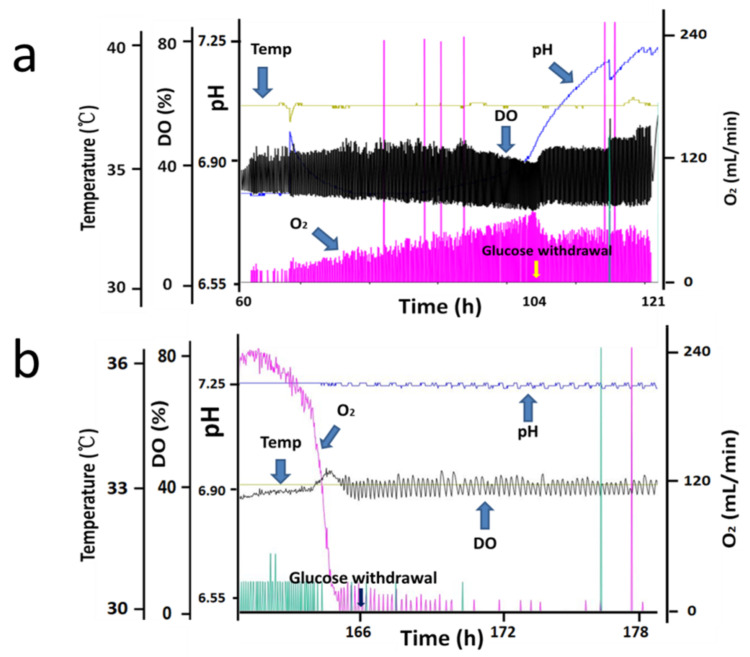
Bioreactor online control curve of process 1 in the exponential and stationary phases (Only one of the three sets of parallel bioreactors on line graphs was shown). The experimental groups stopped adding glucose at day 3 (exponential phase, (**a**)) and day 6 (stationary phase, (**b**)).

Based on changes in cell density, a typical CHO cell fed-batch culture process profile can be divided into two main phases: exponential (growth) phase and stationary (non-growth) phase [24]. The two phases differ significantly in cell metabolism and protein expression [25,26], and a decrease in cell viability was often observed in the late-stationary phase [27,28]; therefore, the effects of glucose withdrawal in two phases of the fed-batch process were studied: the exponential phase and stationary phase. Seven parallel bioreactors were run with process 1, one bioreactor served as the control group, three bioreactors were used to study the effects of glucose withdrawal in exponential phase, and the other three were used to study the effects of glucose withdrawal in stationary phase. The experimental groups stopped adding glucose at day 3 (exponential phase, as shown in Figure 1a) and day 6 (stationary phase, as shown in Figure 1b), respectively, but continued to add FM01A and FM01B to study the effect of glucose withdrawal.

**Figure 2:**
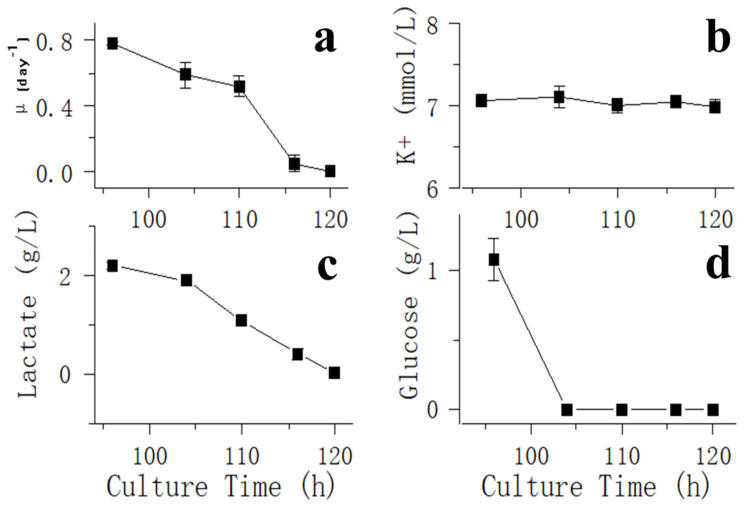
Impact of glucose withdrawal on cell culture performance and metabolite profiles in the exponential phase. Data are expressed as mean ± standard error of three replicates. (**a**) VCD, (**b**) glucose, (**c**) lactate and (**d**) potassium ion (K+) concentrations after day 3 about every 4 h.

**Figure 3:**
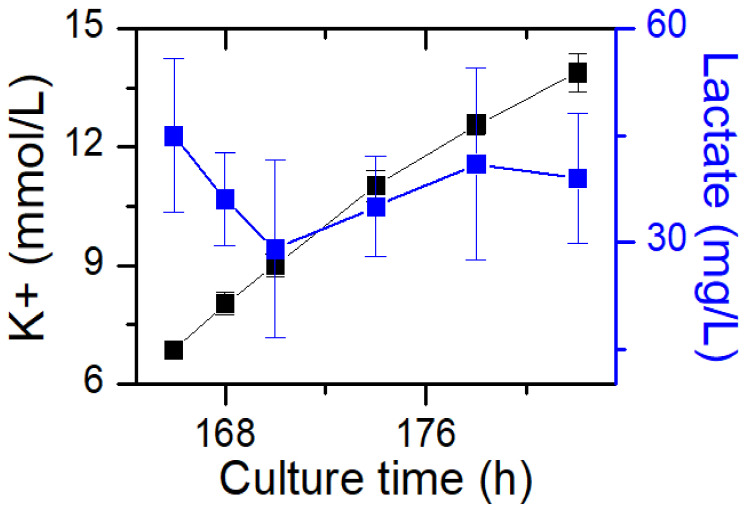
Impact of glucose withdrawal on cell culture metabolite profiles in the stationary phase. Data are expressed as mean ± standard error of three replicates.

For glucose withdrawal in the exponential phase, samples were collected to detect VCD, glucose, lactate and potassium ion (K^+^) concentrations after day 3 about every 4 h (Figure 2). On day 6, when the fed-batch process entered the stationary phase, glucose was not added to bioreactors, and samples were collected to detect VCD, glucose, lactate and K^+^ concentrations about every 4 h (Figure 3).

Figure 2d shows that glucose was depleted at 106 h, and a rapid decrease in oxygen demand was observed (Figure 1a) in the exponential phase. After glucose was consumed, the cells stopped growing (Figure 2a), lactate began to be consumed (Figure 2c) and an increase in pH was observed (Figure 1a). After 121 h, when lactate was only a few dozen mg/L, the oxygen demand dropped to 0, and pH stopped rising. However, amino acids of all types were plentiful at this point (Table 1). After glucose was consumed in the stationary phase, oxygen ventilation was observed to decrease rapidly (Figure 1b), and the lactated content was only 45 mg/L at 166 h. Therefore, 166 h was considered to be the starting point of glucose withdrawal. Then, dissolved oxygen (DO) could be maintained at approximately 40% saturation by intermittently passing a small amount of oxygen (Figure 1b), suggesting that CHO cells almost stopped metabolizing in the absence of the carbon source even when other nutrients, such as amino acids, were sufficient (Table 1).

Lactate was maintained at a low concentration after glucose withdrawal and could not be consumed by cells over time (Figure 3). Lactate has two isoforms: L-lactate and D-lactate. D-lactate is the end molecule of the methylglyoxal pathway and accounts for 10% of total lactate levels [29]. Unlike L-lactate, D-lactate cannot be re-utilized by CHO cells. Therefore, the small amount of unutilized lactate remaining in process 1 was likely to be D-Lactate.

Further, the consumption rate of amino acids were analyzed after glucose withdrawal (Table 1), and the result showed that the total amino acid consumption rate of the experimental group was faster than that of the control group in the experimental phase. In the stationary phase, CHO cells were inefficient in utilizing most of amino acids, except for asparagine, glutamine and alanine, after there was no glucose or other available carbon source. This may be due to the lack of carbon sources, resulting in an insufficient production of ATP, which could not support the operation of other functions of CHO cells. This result also showed that amino acids were not a good carbon source for CHO cells.

**Figure 4:**
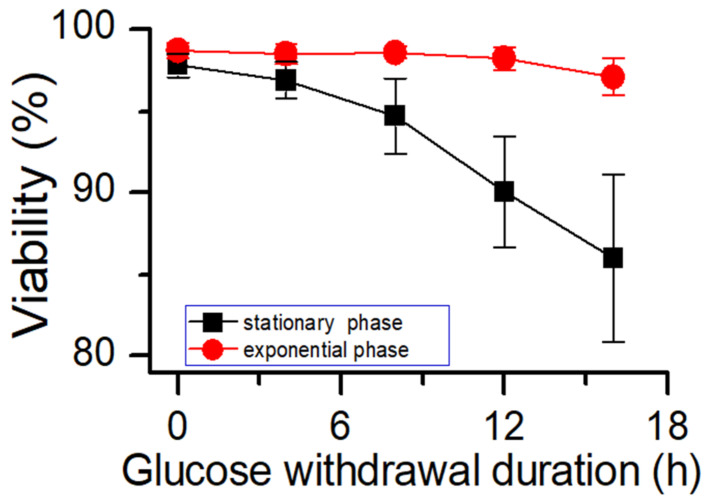
Effects of glucose starvation on cell viability in different culture phases. Data are expressed as mean ± standard error of three replicates.

**Figure 5:**
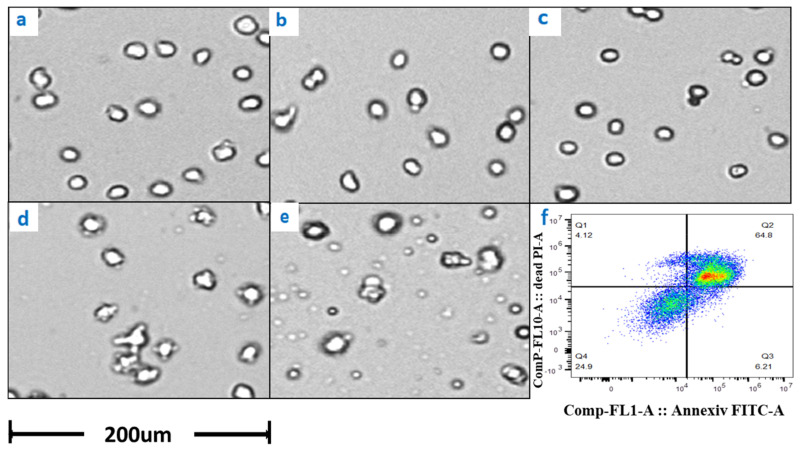
Changes in cell morphology with glucose withdrawal for (**a**) 0, (**b**) 4, (**c**) 8, (**d**) 12, and (**e**) 16 h in stationary phase. (**f**) represents the flow cytometry results of glucose withdrawal for 16 h, based on Annexin V marker.

**Figure 6:**
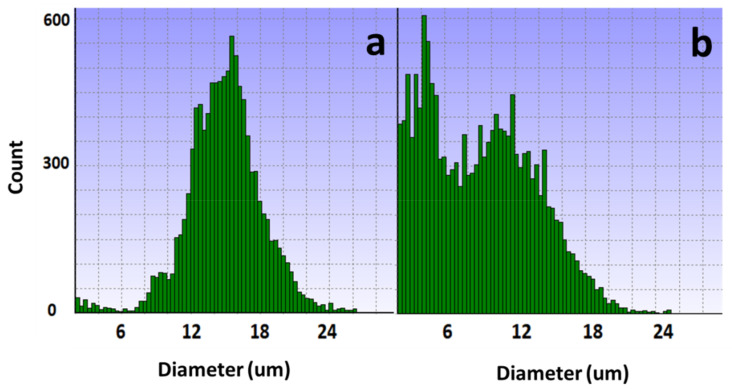
Changes in cell diameter with glucose withdrawal for (**a**) 0 and (**b**) 16 h.

The results of potassium ion concentration (Figure 2b and Figure 3) showed that after glucose was depleted, the potassium concentration did not change in the exponential phase but increased in the stationary phase. This required further exploration of the reasons. So, cell viability (Figure 4) and morphology (Figure 5) were detected. In the exponential phase, the cell viability (Figure 4) and morphology (not shown) were not affected by glucose withdrawal because there was some lactate in the exponential phase, which could be used as carbon source for CHO cells. In the stationary phase, with the prolongation of glucose starvation time, the cell viability decreased (Figure 5). Further, cell membranes showed blebs, and cells of smaller diameters were produced. At the beginning of glucose withdrawal, the diameter of CHO cells was mainly 12–20 µm, but after 16 h of glucose withdrawal, abundant cells with a diameter <10 µm were produced (Figure 6). The results of flow cytometry analysis showed that there was a large amount of apoptosis (Figure 5f). Grilo et al. reported that three main cell death pathways existed in all industrially relevant cell lines: apoptosis, autophagy, and necrosis [30]. Apoptosis is responsible for 80% of CHO cell deaths and could cause cell membranes to form blebs and produce apoptotic bodies ranging from 50 to 5000 nm in diameter [31,32]. Kakarla has reported that apoptosis produces extracellular vesicles such as apoptotic bodies, micro-vesicles and exosomes at the same time [33]. Exosomes are now considered to be extracellular vesicles, 50–100 nm in size that are released by most or all somatic cells and perform more functions than garbage disposal [34]. So, based on these previous studies, the vesicles in Figure 5 were apoptotic bodies of CHO cells. Tang et al. reported that tyrosine starvation induced cell autophagy, and pH decreased in fed-batch cultures [35]. Necrosis could cause cell volume increase and cell membrane permeabilization and rupture [36]. Therefore, according to the findings of this study, the main mode of cell death caused by glucose withdrawal was apoptosis, and the diameter of apoptotic bodies produced by CHO cells was mainly below 10 µm. The proportion of apoptotic bodies with a diameter of approximately 3 µm was the highest (Figure 6b). Five main apoptosis pathways have been identified [30], and proteomic studies on CHO cells indicate that the intrinsic pathway is the most relevant one [37]. The intrinsic apoptosis pathway can be directly triggered by nutrient deficiency, excess temperature, or some other inappropriate culture conditions.

Figure 3 shows that in the stationary phase, the potassium ion concentration increases with the prolongation of glucose withdrawal time, but this result was not found in the exponential phase (Figure 2). Combined with the results of Figure 4 and Figure 5, it was suggested that the increase of extracellular potassium ion concentration may be related to apoptosis. In the exponential phase, due to the presence of lactate, the cells could continue to maintain their physiological function after glucose depletion, and no apoptosis occurred. In the stationary phase, after glucose depletion, the cells had no available carbon source, so cell apoptosis occurred. Pigozzi et al. reported that a prolonged efflux of potassium ions from the cell could accelerate the apoptosis process [38]. Therefore, we speculate that the efflux of potassium ions was related to apoptosis. So, the detection of potassium ion concentration during cell culture process may be important, and an abnormal potassium ion concentration increase may indicate the onset of apoptosis.

### 3.2. Effect of Apoptosis on CHO Cells

**Figure 7:**
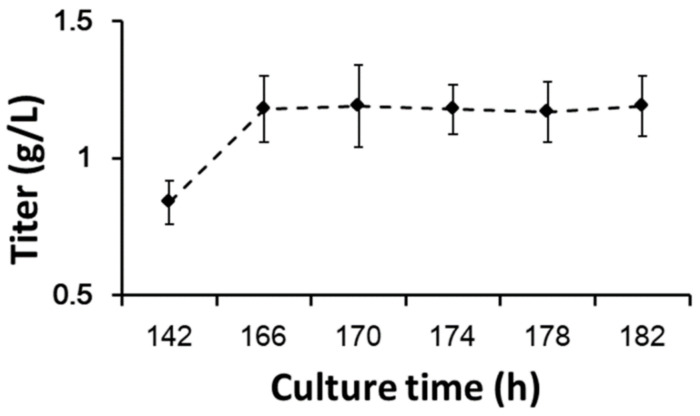
Protein yields with prolonged glucose withdrawal. Data are expressed as mean ± standard error of three replicates.

Apoptosis may affect process properties and product quality [39], so further studies are needed. The protein yields of different timepoints of glucose withdrawal at the stationary phase are shown in Figure 7. CHO cells stopped protein expression immediately after glucose withdrawal. This result was comparable to the cellular oxygen demand in process 1 in the stationary phase (Figure 1b). Protein synthesis required abundant ATPs, so when glucose and other carbon sources were withdrawn, cells could not synthesize ATPs.

**Figure 8:**
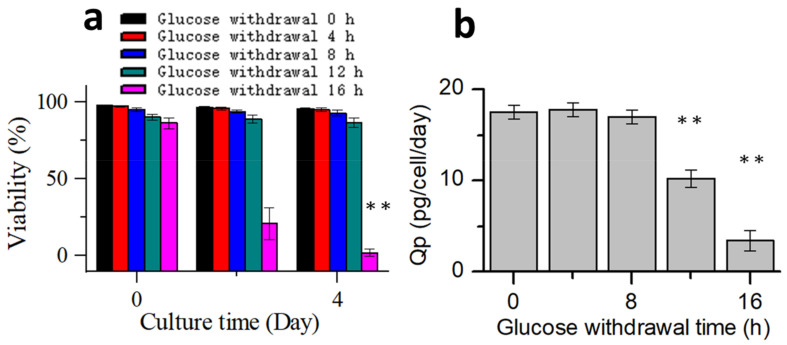
Process performances after glucose supplementation. (**a**) Change in the cell viability with culture time. (**b**) Effect of different glucose withdrawal times on the specific productivity of the protein expression. Data are expressed as mean ± standard error of three replicates. ** *p* < 0.01.

Cells were collected from the bioreactors at 0, 4, 8, 12, and 16 h after glucose withdrawal in the stationary phase and then placed in 250 mL shake flasks to continue culturing, and glucose was added. Five groups of shake flasks continued to be cultured according to the process 1 for 4 days. Samples were taken out once every two days to detect the cell viability (Figure 8a). Within 12 h of glucose withdrawal, the subsequent cell viability was not significantly affected. However, glucose withdrawal for 16 h significantly affected the cell viability in the continued culture. Subsequently, the samples were used for titer and physicochemical analyses. Specific productivity (Qp) was calculated (Figure 8b) [40]. The Qp of the protein expression did not change significantly after 8 h of glucose withdrawal but decreased after 12 h of glucose withdrawal. The results of antibody quality analysis showed that apoptosis had no significant effect on the size variant, charge variant, or oligosaccharide profile (Table 2). These results indicated that during the stationary phase, CHO cells could tolerate glucose withdrawal for at least 8 h without significantly affecting the antibody expression or quality. During glucose withdrawal, cells stopped almost all metabolisms, but when glucose was replenished, cell metabolisms were re-activated. To the best of our knowledge, this is the first study to report that CHO cells can tolerate prolonged glucose withdrawal without affecting protein expression or quality. This result could serve as a reference for harnessing glucose withdrawal to control the lactate metabolism in industrial cell cultures.

The harvest material was purified via protein A chromatography. Protein A eluates from the fed-batch shake flask experiments were tested for impurities via Size Exclusion Chromatography (SEC), charge heterogeneity via Cation exchange Chromatography (CEX), N-glycan profile viaUPLC-FLD, and SA via HPLC-FLD. The values of each parameter are reported as average ± standard error (*n* = 3).

**Figure 9:**
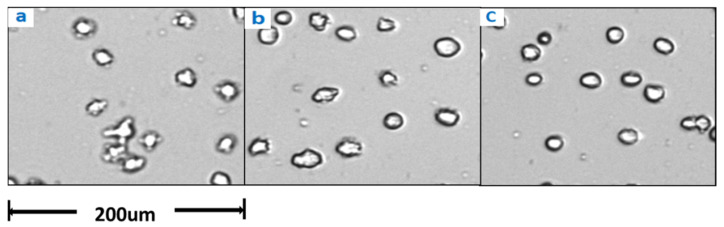
Changes in cell morphology after glucose withdrawal for 12 h (**a**) before glucose supplementation, (**b**) 24 h after glucose supplementation, and (**c**) 48 h after glucose supplementation.

CHO cells were sampled from the bioreactor when glucose was withdrawn for 12 h and then supplemented with glucose to continue the culture (Figure 9). With prolonged culture time, apoptosis alleviated, indicating that early apoptosis was reversible. Therefore, apoptosis needs to be paid attention to at all times, and the process can be adjusted to reverse the early apoptosis.

### 3.3. Process Robustness and Glucose Withdrawal

**Figure 10:**
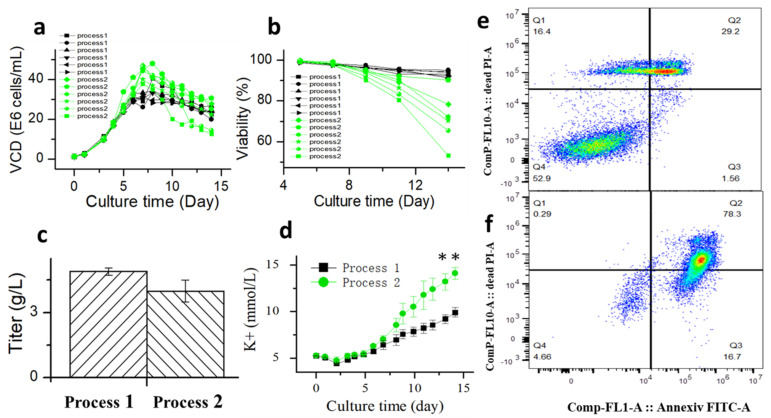
Comparison of viable cell density (**a**), cell viability (**b**), titer (**c**), extracellular potassium concentration (**d**), cell apoptosis on D14 of process 1 (**e**) and cell apoptosis on D14 of process 2 (**f**) based on Annexin V marker. Data are expressed as mean ± standard error of six replicates. ** *p* < 0.01.

The fed-batch process may affect the ability of CHO cells to tolerate glucose withdrawal, and a robustness process may improve the ability of CHO cells to tolerate apoptosis. Therefore, glucose withdrawal was studied in process 2. Processes 1 and 2 differed in incubation temperature. Process 1 was shifted to 33 °C on day 5 of cultivation, while process 2 was maintained at 36.5 °C. Culture temperature shifts to a lower value are frequently used to improve the viability, productivity, or protein quality in CHO cell cultures [41,42]. The results of comparing six batches of historical data of process 1 and process 2 showed that process 1 had better robustness compared to process 2 (Figure 10a,b). Further comparing the extracellular potassium ion concentration of the two processes, the results showed that the extracellular potassium ion concentration of process 2 was significantly higher than that of process 1 (Figure 10c). These results were comparable to the apoptosis results of process 1 (Figure 10e) and process 2 (Figure 10f), which further suggested that there was a positive correlation between extracellular potassium ion concentration and cell apoptosis.

**Figure 11:**
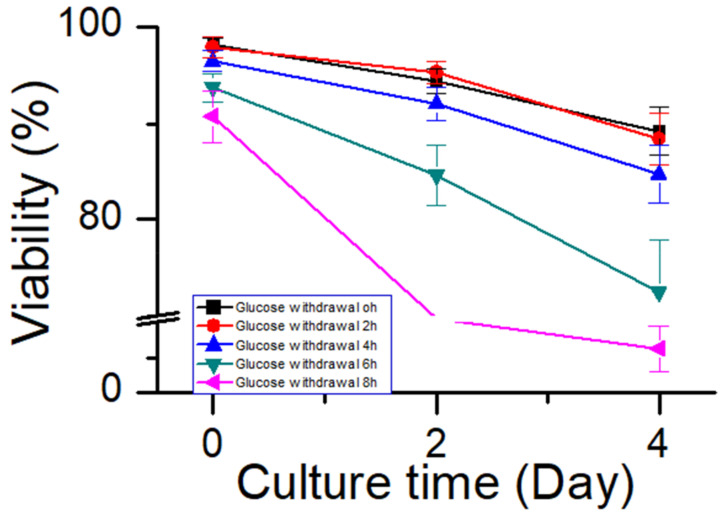
Effect of glucose withdrawal on cell viability in process 2. Data are expressed as mean ± standard error of three replicates.

To further investigate the relationship between the ability of CHO cells to tolerate apoptosis and the process stability, glucose supplementation was stopped when cells of process 2 were cultured to day 6. The timepoint at which the oxygen demand dropped to almost 0 was considered to be the onset of glucose withdrawal. Subsequently, the samples were collected every 2 h and placed inside shake flasks. After supplementing glucose, the cells continued to be cultured according to process 2. The samples were collected after 2 and 4 days of culture to test the cell viability (Figure 11). After 6 h of glucose withdrawal for process 2, and the cell viability was affected significantly. However, in process 1 (Figure 8a), the cell viability was unaffected within 8 h of glucose withdrawal. These results suggested that process robustness affected the ability of cells to tolerate glucose withdrawal and that a stable process (process 1) enabled cells to tolerate glucose withdrawal for longer periods. This result may serve as a reference for cell culture engineers to study process robustness using the ability of cells to tolerate apoptosis. Process characterization studies could be used to demonstrate the process robustness of the cell culture [10,11], but they have the requirements of long study duration, high personnel numbers, and good equipment. Therefore, an easy method is required to judge the robustness of the process during cell culture process development. This glucose withdrawal study was easy to perform to reduce apoptosis; therefore, it was suitable for the preliminary judgment of the process stability.

## 4. Discussion

Apoptosis is responsible for 80% of Chinese hamster ovary (CHO) cell deaths and affects the robustness of the cell culture process. Therefore, we need to pay great attention to the occurrence of apoptosis in process development. The results of this study showed that glucose withdrawal could be used as an effective method to study the apoptosis of CHO cells. After the depletion of available carbon sources, oxygen consumption by CHO cells rapidly reduced to almost 0 (Figure 1), and the cells entered a state similar to animal hibernation. Subsequently, the cells also stopped protein expression. Cells underwent apoptosis with prolonged glucose withdrawal, consistent with previous studies [31,32]. Furthermore, CHO cells produced a large number of apoptotic bodies after apoptosis (Figure 5), and their diameters were mainly distributed near 3µm (Figure 6), and through flow cytometry analysis, a large number of apoptosis was found. This was also the first direct proof that the cause of cell death from glucose withdrawal was apoptosis, and apoptotic bodies can be used as a morphological indicator of apoptosis in CHO cells. Apoptosis can significantly reduce the expression of protein, but it is reversible in the early stage (Figure 9). So, it is necessary to detect apoptosis early to reduce its effect on protein expression. This study results showed that apoptosis did not affect the quality of proteins (Table 2).

At present, the detection of apoptosis has not been widely used in large-scale cell cultures. Therefore, a fast and simple method for detecting apoptosis is needed. These study results showed that the apoptosis of CHO cells was accompanied by an increase in extracellular potassium concentration, which was thought to play a role in accelerating apoptosis [38]. A further comparison of historical data (Figure 10) showed that there was a positive correlation between potassium concentration and apoptosis. Therefore, the concentration of extracellular potassium ions could be used as a biochemical detection method to characterize apoptosis and the robustness of the process. Furthermore, these results may be used in the diagnosis and treatment of diseases [43].

Currently, the assessment of process robustness is often time-consuming, labor-intensive, and material-intensive using process characterization studies. Therefore, a simple and time-saving method is highly needed for the biopharmaceutical industry. Previous studies showed that the CHO cell metabolism varies greatly across phases and processes [24,44], and different culture phases and processes resulted in different abilities of CHO cells that tolerated glucose withdrawal. Furthermore, this study showed that a more robust process (process 1) allows CHO cells to tolerate apoptosis for a longer period. This indicates that there was a correlation between apoptosis and the robustness of the process. This method should be validated by more future studies.

## 5. Conclusions

Apoptosis could significantly affect the performance and robustness of the cell culture process, so it needs more attention. Based on the results of this study, apoptotic bodies can be used as morphological indicators of apoptosis, and extracellular potassium concentration can be used as a biochemical indicator of apoptosis in CHO cells. Furthermore, the ability of cells to tolerate apoptosis in the process may be related to the robustness of the process, which allows cells to tolerate apoptosis for a longer time. So, apoptosis may be a rapid method to judge the robustness of the process, but more research data are needed to confirm this.

## Figures and Tables

**Table 1:** Specific consumption rate of amino acids (nmol/10^6^ cells/h).

	Exponential Phase		Stationary Phase	
	Glucose Withdrawal	Glucose Sufficient	Glucose Withdrawal	Glucose Sufficient
Aspartate	−3.1 ± 0.9	−1.8	0.1 ± 0.5	−1.5
Glutamate	−5.5 ± 1.2	−3.3	0.2 ± 0.3	−2.8
Serine	−3.0 ± 0.9	−3.4	−0.1 ± 0.2	−2.9
Asparagine	−4.3 ± 0.3	−2.1	−2.0 ± 0.7	−1.6
Glutamine	−0.3 ± 1.8	0.1	−0.7 ± 0.4	0.0
Histidine	−0.7+0.2	−0.5	0.3 ± 0.2	−0.4
Glycine	−0.9 ± 0.5	1.3	4.7 ± 0.5	0.7
Threonine	−3.0 ± 1.3	−1.9	1.4 ± 0.7	−1.7
Arginine	−0.9 ± 0.8	−0.9	0.5 ± 0.4	−0.8
Alanine	−5.4 ± 2.1	−4.9	−2.0 ± 0.6	−0.7
Tyrosine	−1.3 ± 1.1	−1.1	0.7 ± 0.5	−1.0
Cysteine	−0.9 ± 0.2	−1.0	1.0 ± 0.7	−0.7
Valine	−3.1 ± 0.7	−3.1	0.8 ± 0.5	−2.3
Methionine	0.1 ± 1.5	−0.5	1.2 ± 0.7	−0.4
Tryptophan	−0.7 ± 0.4	−0.4	0.2 ± 0.4	−0.4
Phenylalanine	−1.4 ± 0.5	−1.0	0.6 ± 0.5	−0.8
Isoleucine	−2.9 ± 0.7	−1.4	0.6 ± 0.4	−1.1
Leucine	−4.5 ± 1.0	−2.5	0.7 ± 0.6	−2.0
Lysine	−1.5 ± 0.3	−1.8	0.5 ± 0.4	−1.5

Values are reported as the average ± standard error (*n* = 3).

**Table 2:** Effect of apoptosis on the quality of antibody.

	SEC Purity (%)	CE-NR Purity (%)	Charge Heterogeneity			N-Glycan Profile			
			Acidic (%)	Main (%)	Basic (%)	G0 (%)	G0F (%)	G1F (%)	G2F (%)
Control	99.21 ± 0.23	96.14 ± 0.95	20.37 ± 0.53	74.97 ± 0.61	4.66 ± 0.26	3.87 ± 0.37	85.11 ± 0.89	7.02 ± 0.60	0.44 ± 0.07
Apotosis	99.25 ± 0.17	95.71 ± 0.84	19.08 ± 0.29	75.70 ± 0.34	5.13 ± 0.07	3.99 ± 0.41	86.05 ± 0.84	6.47 ± 1.42	0.35 ± 0.1

## Data Availability

All supporting data are included within the article.
